# The Association Of Serum Cortisol Level With Microalbuminuria In Patients With Type 2 Diabetes And Prediabetes

**DOI:** 10.7150/ijms.48742

**Published:** 2020-10-18

**Authors:** Xiaodan Zhang, Xiaoyi Deng, Jianlong Zhou, Kangshou Qiu, Mingye Deng, Zhuohang Lin, Singla Sethiel Mosha, Wangen Li

**Affiliations:** 1 Department of Endocrinology, The Second Affiliated Hospital of Guangzhou Medical University, 250 East Changgang Road, Haizhu District, Guangzhou, China; 2 The Second Clinical Medicine School, Guangzhou Medical University, Guangzhou, China

**Keywords:** Hydrocortisone, Diabetes Mellitus, Type 2, Prediabetic State, Diabetic Nephropathies

## Abstract

Whether cortisol secretion is linked with microalbuminuria remains undefined. We aimed to investigate the relationship between serum cortisol levels and the presence of microalbuminuria in patients with type 2 diabetes (T2DM) and prediabetes. A cross-sectional study was conducted with 211 patients with T2DM or prediabetes. Serum cortisol was measured at 8:00 h, 16:00 h, and 0:00 h. The level and circadian rhythm of ACTH were also evaluated. Urine excretion of albumin was measured. Patients were subdivided into microalbuminuria (MAU) group (n= 120) and normoalbuminuria (NAU) group (n = 91) according to the status of microalbuminuria. Levels of serum cortisol (8:00 h: 426.9 ± 155.0 nmol/; 16:00 h: 303.7 ± 144.7 nmol/L) were significantly higher in MAU group than in NAU group (8:00 h: 370.2 ±130.6 nmol/L, *P* = 0.004; 16:00 h: 234.7 ± 120.2 nmol/L, *P* = 0.001). After adjustment for multiple factors, the correlation between cortisol levels (both at 8:00 h (*P* = 0.005) and at 16:00 h (*P* = 0.001)) and microalbuminuria remained consistent and significant. Higher levels of cortisol (cut-off value: 390.5 nmol/L at 8:00 h, 203.5 nmol/L at 16:00 h) help to detect the development of microalbuminuria. Serum cortisol secretion is associated with the presence of microalbuminuria in patients with T2DM and patients with prediabetes. Higher levels of cortisol, even in the normal range, may be related with the development of microalbuminuria.

## 1. Introduction

Cortisol, a kind of glucocorticoid hormone, plays important roles in many metabolic processes. Environmental factors and some prevalent non-communicable diseases such as obesity can disturb the cortisol regulatory and effector pathways 1. Hypercortisolism is linked to various diseases, including diabetes, obesity, hypertension, dyslipidemia, osteoporosis, arteriosclerosis, and cardiovascular disease 2, 3, 4. The prevalence of hypercortisolism is higher in patients with diabetes than in the general population 5. Cortisol level was found to be positively associated with glycated hemoglobulin (HbA1c), independent of diabetes medication use 6. However, results concerning the relationship of cortisol and cardiometabolic components were inconsistent. Some studies showed negative or opposite results, especially when recruiting patients with lower BMI values rather than overweight or obese patients 7, 8. Therefore, we consider it necessary to evaluate the hypothalamic-pituitary-adrenal axis (HPA) activity in patients with a broader range of BMI values who were diagnosed with type 2 diabetes (T2DM) or prediabetes.

Besides the traditional cardiometabolic factors, cortisol secretion is also suggested to correlate with renal function in a few studies 9, 10. Serum cortisol level was negatively correlated with eGFR levels in patients with essential hypertension. Non-suppressing cortisol with a low-dose dexamethasone suppression test was found to be associated with the presence of microalbuminuria in patients with metabolic syndrome (MetS) 10. But evidence is lacking on this topic in diabetic patients, In this study, we aimed to evaluate the HPA activity and the urinary albumin levels, so as to determine whether there is an association of cortisol secretion with microalbuminuria.

## 2. Materials and Methods

*2.1. Study population.* Data were collected retrospectively from January 2016 to September 2019 at The Second Affiliated Hospital of Guangzhou Medical University. Selection criteria were hospitalized patients with diagnosed T2DM, impaired glucose tolerance, or impaired fasting glucose. Exclusion criteria were as follows; A. diagnosed adrenal diseases including Cushing syndrome, pheochromocytoma, and primary hyperaldosteronism; B. with history of surgery of the central nervous system; C. with history of pituitary disease; D. severe infection; E. severe stress including acute myocardial infarction and diabetic ketoacidosis; F. application of glucocorticoids in the last 3 months; G. severe impaired liver function; H. history of malignant tumors; I. severe hypoglycemia during the last 3 months; J. severe dysregulation of serum electrolytes. If the serum cortisol level at 8:00 h exceeded the upper limit of normal range, an overnight dexamethasone (1 mg) test was done to exclude subclinical Cushing's syndrome. Sufficient suppression of serum cortisol was defined as < 50 nmol/L. Eventually, a total of 211 patients were involved for assessment. This study was approved by the Ethics Committee of the Second Affiliated Hospital of Guangzhou University. The committee waived the need for informed consent due to the retrospective nature of the study with no impact on health outcome.

*2.2. Measurement and data collection.* Demographic information including history of prescription medication usage and habits of smoking was collected through review of medical records. Body height and weight was measured, and the body mass index (BMI) was calculated. Blood pressure (BP) was measured twice, in a sitting position, on the right upper arm in the line with the heart after a minimum rest period of 10 minutes. Diabetes, impaired glucose tolerance, impaired fasting glucose, and impaired glucose regulation were defined in accordance with the 1999 World Health Organization (WHO) criteria. Patients on medication for diabetes were also defined as having diabetes. Concentrations of serum cortisol and ACTH at 8:00 h, 16:00 h, and 0:00 h were determined using chemiluminescent enzyme immunoassay (IMMULITE 2000 ACTH and IMMULITE2000 Cortisol, Siemens Healthcare Diagnositics Inc., CA, USA). Normal ranges were as follows: cortisol (8:00 h) 170-440 nmol/L, cortisol (16:00 h) 60-250 nmol/L, cortisol (0:00 h) 55-138 nmol/L, and ACTH (8:00 h) 0-10.21 pmol/L. Disturbed circadian rhythm of cortisol was defined if the level of cortisol at 8:00 h was lower than the one at 16:00 h or 0:00 h. Venous blood samples were collected in the morning after an overnight fast for laboratory measurement of other parameters. Routine biochemical parameters (including fasting plasma glucose (FPG), 2h postprandial plasma glucose (2h PG), C-Peptide, 2h postprandial C-Peptide, glycated hemoglobin (HbA1c), serum high-density lipoprotein cholesterol (HDL-C), low-density lipoprotein cholesterol (LDL-C), total cholesterol, triglyceride, Apolipoprotein A1 (ApoA1), Apolipoprotein B (ApoB), uric acid, serum creatinine, high sensitivity C-reactive protein (hs-CRP), and albumin) were measured by routine laboratory methods. The estimated glomerular filtration rate (eGFR) was calculated using Modification of Diet in Renal Disease equation: eGFR (mL/min/1.73 m^2^) = 186 × (SCr/88.4)^-1.154^×(age)^-0.203^ × (0.742 if female) 11. Urine samples of 24 hours were collected to measure urine albumin levels using a chemiluminescence assay. Spot urinary samples of patients were collected at 7:00-8:00 am. Urinary albumin levels were measured from spot samples using nephelometry immunoassay and urinary creatinine levels were measured using velocity method. The urinary albumin-to-creatinine ratio (UACR) was calculated. Microalbuminuria was defined as a UACR of 30-300 mg/g or an albumin excretion rate (AER) of 30-300 mg/24 hours.

*2.3. Statistical analysis.* Numeric values were presented as mean (standard deviation) or median (interquartile range). Categorical values were presented as number (%). Comparisons for continuous variables were performed using Student's t-test for parametric variables and Mann-Whitney U test for non-parametric variables. Categorical variables were compared using Chi-square test. The associations between variables were evaluated by Spearman correlation coefficient test. Correlations between microalbuminuria and cortisol levels were analyzed with multivariate logistic regression after adjusting for possible confounding factors found by univariate regression analysis. Receiver operating characteristic (ROC) curves were constructed to determine cut-off values of serum cortisol for predicting the presence of microalbuminuria. Confidence intervals (CIs) of 95% were used. A *P*-value < 0.05 was considered statistically significant. All statistical analyses were performed using IBM SPSS statistical software version 22 for Windows (IBM Corp., Armonk, New York, USA).

## 3. Results

*3.1. Clinical characteristics of the study subjects.* Baseline and clinical characteristics of the enrolled subjects are presented in Table 1. Most patients (n=198) were diagnosed with T2DM. Thirteen patients were diagnosed with prediabetes (IGT n=12, IFG n=1). The median age of the subjects was 56.0 years old, ranging from 17 to 90 years, and 53.1% of patients were female. Median duration of diabetes was 3.0 years (range 0-30 years). The median BMI of the patients was 27.0 kg/m^2^ (range 17.5-47.1 kg/m^2^). About 70% of the study population had a past history of hypertension. Microalbuminuria was found in 91 patients (43.1%). Subjects were allocated to normoalbuminuria (NAU) group (n = 120) or microalbuminuria (MAU) group (n = 91). The prevalence of hypertension and diabetic retinopathy was significantly higher in patients with microalbuminuria. Levels of serum cortisol (both at 8:00 h and at 16:00 h), BP (both systolic and diastolic) and high sensitivity C-reactive protein (hsCRP) were significantly higher, while eGFR levels were significantly lower in the MAU group than in the NAU group.

*3.2. Analysis of associated factors of microalbuminuria.* Correlations between clinical characteristics and microalbuminuria are presented in Table 2. Univariate regression analysis revealed associated factors of microalbuminuria including past history of hypertension, presence of diabetic retinopathy, both systolic and diastolic BP, eGFR and serum cortisol at 8:00 h and cortisol at 16:00 h. Furthermore, after adjustment for multiple factors (presence of diabetic retinopathy, systolic and diastolic BP, HbA1c, and smoking status), the correlation between cortisol levels (both at 8:00 h and at 16:00 h) and microalbuminuria remained consistent and significant. Spearman correlation coefficient tests were also performed to investigate the relationship of quantified urinary protein levels and clinical factors. In accordance with the result of regression analysis, the correlation tests revealed associated factors including past history of hypertension, smoking, presence of diabetic retinopathy, HbA1c, both systolic and diastolic BP, eGFR and serum cortisol at 16:00 h (Table 3). Cortisol levels (both at 8:00 h and at 16:00 h) were found to be related with urinary albumin levels (See Supplementary Materials).

**Figure 1:**
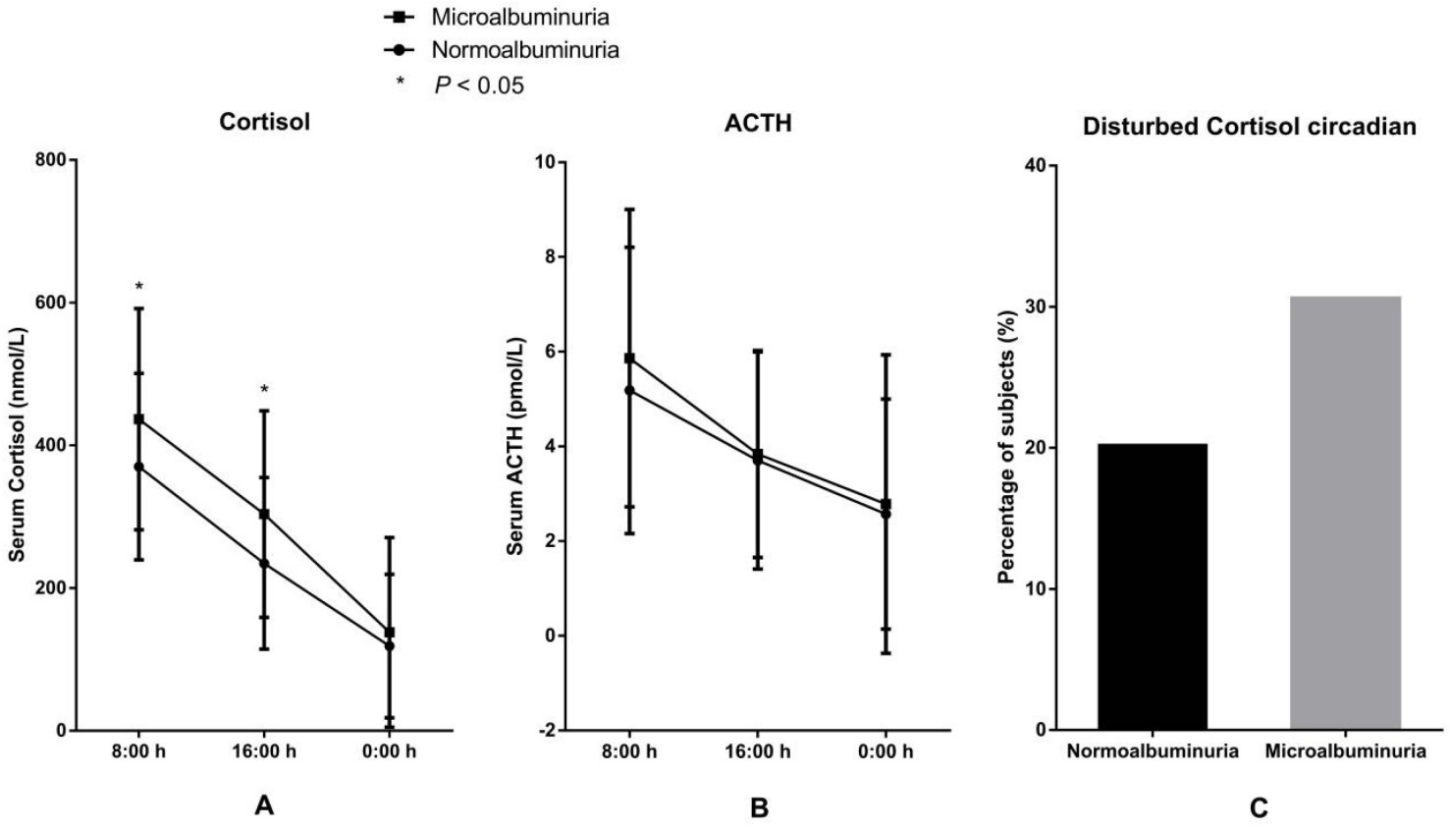
Levels and circadian rhythm of serum cortisol and ACTH in patients with microalbuminuria and normoalbuminuria. A. Levels of serum cortisol. B. Levels of ACTH. C Proportions of patients with disturbed cortisol circadian rhythm.

*3.3 Correlation between serum cortisol and microalbuminuria.* The levels and circadian rhythm of serum cortisol and ACTH in the two groups are presented in Figure 1. Levels of serum cortisol both at 8:00 h and at 16:00 h were significantly higher in the MAU group than in the NAU group. The proportion of patients with disturbed circadian rhythm of cortisol, the levels of ACTH (8:00 h, 16:00 h, and 0:00 h) and the level of cortisol at 0:00 h were also higher in the MAU group than in the NAU group, but the differences were not statistically significant.

**Figure 2:**
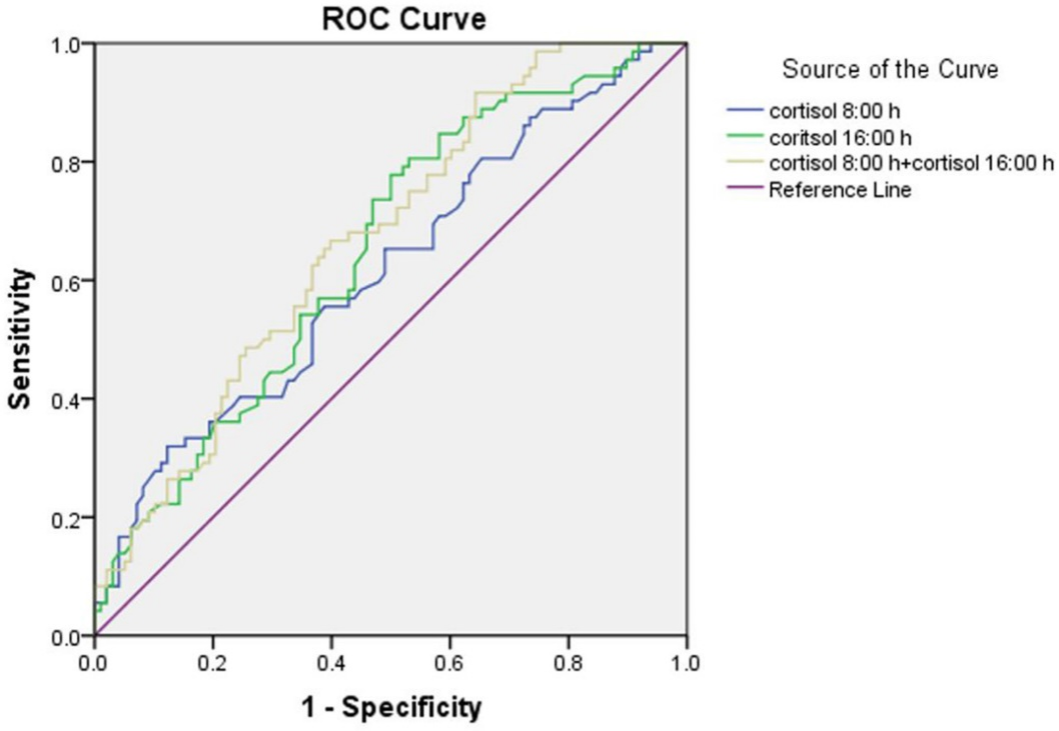
Receiver operator characteristic (ROC) curve of serum cortisol at 8:00 h and at 16:00 h for detecting microalbuminuria.

The results of the ROC curve analysis are presented in Figure 2. The discriminatory power of the combination of cortisol at 8:00 h and at 16:00 h (area under the curve (AUC) = 0.670 ± 0.041) for microalbuminuria was stronger than the individual ones. The optimum cortisol cut-off for detecting the presence of microalbuminuria was 390.5 nmol/L (58.2% sensitivity, 58.3% specificity, and AUC = 0.615 ± 0.044) for cortisol at 8:00 h and 203.5 nmol/L (77.8% sensitivity, 50.0% specificity, and AUC = 0.648 ± 0.042) for cortisol at 16:00 h.

## 4. Discussion

Microalbuminuria is an early manifestation of diabetic nephropathy and may be indicative of diffuse endothelial disease. Prediabetes and T2DM are associated with generalized microvascular dysfunction, including microalbuminuria and reduced kidney function 12, 13. The present study demonstrated a relationship of increased serum cortisol and the presence of microalbuminuria in subjects with T2DM or prediabetes. Previously, a study showed that patients with MetS and disturbed cortisol balance display more microalbuminuria 10. In patients with T2DM, increased hypothalamic-pituitary-adrenal axis (HPA) activity was found to be related to the presence of some diabetic chronic complications, and the level of cortisol secretion was linked with the number of diabetic complications. But an association between the presence of diabetic nephropathy and cortisol levels was not found 14, which was different from our results.

Cortisol concentrations, evaluated in serum, hair or salivatory forms, were considered to be related to many metabolic features in patients with metabolism-related disorders, such as diabetes, obesity, and MetS 15-19. Higher glycemic levels, increased insulin resistance, and decreased β-cell function were most commonly reported to be correlated with higher cortisol levels 15, 18, 20. A previous study even showed higher odds of T2DM in patients with higher cortisol 15. Higher level of cortisol after 1mg-overnight-dexamethasone was associated with increased risk of T2DM, hypertension and fragility fractures 21. Nevertheless, the relationship of cortisol with cardiometabolic factors or results varied across studies. More recently, a prospective study in children in mid-childhood indicated that hair cortisol concentration was not associated with cardiometabolic biomarkers except for a slight increase in insulin resistance 22. A study including a large group of healthy adults showed that neither basal nor evening salivatory cortisol was associated with incident insulin resistance or T2DM 7. The differences in outcome could partly be explained by the varied BMI values across the studies (BMI, 31.7 ± 7.2 kg/m^2^ 13; BMI, 20.2 ± 3.7 22; BMI, 25.4 ± 4.1 kg/m^2^ 7). Weight seemed to have an important impact on cortisol levels. Serum cortisol was reported to be responsive to weight change. Effective weight loss by lifestyle intervention and bariatric surgery were both associated with decreased cortisol levels in obese individuals 20, 24. Among subjects with similar BMI values, no significant difference was found in cortisol levels between patients with MetS and those without MetS 9. Also, varied cortisol secretion patterns might present in patients in different age or development phase. A study in children and adolescents showed that overweight and obese was not associated with elevated hair cortisol concentration 25. In the present study, serum cortisol levels (8:00 h and 16:00 h) were associated with some of the glycemic variables (FPG, HbA1c). Except for glycemic parameters, no significant relationship between cortisol levels with BMI or other metabolic variables was indicated. The median BMI value of patients in this research was 27.0, which was higher than the healthy population but was lower than most studies investigating the level and impact of cortisol. This may partly explain the negative correlation findings.

Previously, only a few studies investigated the relationship of cortisol and renal function. A study in patients with essential hypertension demonstrated that serum cortisol level was negatively correlated with eGFR levels. But the researchers did not assess proteinuria 9. In the present study, we found a significant positive correlation between serum cortisol and microalbuminuria, but not eGFR. This is in accordance with the varied pathogenesis and early manifestation of kidney lesion in diabetes and hypertension. Due to the cross-sectional nature of the study, definite cause-and-effect relationship between serum cortisol and microalbuminuria could not be established. But concerned with the well-recognized deleterious effects of glucocorticoids on glucose metabolism, it is reasonable to speculate that increased cortisol secretion may contribute to the progression of diabetes and the development of microalbuminuria. In the present study, no significant difference was found in most metabolic parameters, including those reflecting glycemic levels, β-cell function or insulin resistance or demographic characteristics such as age of patient or duration of diabetes between patients with microalbuminuria and those without microalbuminuria. This indicates that there might be other working mechanisms involved except for worsened glycemic control for the development of microalbuminuria. On the other hand, cortisol, as a stress-induced hormone, may be increased in diabetic subjects with chronic complications who were suggested to be exposed to chronic stress 26. In this regard, further explorations are needed to determine whether there is a causality or interaction relationship between cortisol and microalbuminuria. Although only an association could be established so far, the study showed that a higher level of cortisol, even in the normal range, may be related with the development of microalbuminuria. Moreover, a combination test of cortisol levels in two different time points, one in the morning and one in the afternoon, increase the discriminatory power. Most studies focused on morning or midnight cortisol secretion, and cortisol levels in the afternoon were rarely concerned. Our results suggest that cortisol level in the afternoon is also worthy of attention and may indicate some important metabolic changes. Furthermore, dynamic testing of daytime cortisol levels has stronger discriminatory power than testing at a single time-point. No significant association was found between cortisol secretion and the presence of other diabetic complications including retinopathy, peripheral neuropathy, or foot ulcer in our study. This might be explained by the low prevalence of these complications and the limited sample size.

There are several limitations of the present study. First, as mentioned above, only a limited number of subjects in a single center was involved. Also, the samples were derived from an inpatient setting, which might lead to a condition of enhanced HPA activity and thus cause evaluation bias 27. Second, all the outcomes were achieved by retrospective analysis, which does not allow us to draw conclusions about causality. Third, only a small group of patients with prediabetes was included. In this regard, though no significant difference was found in the prevalence of microalbuminuria between patients with T2DM and patients with prediabetes, the results of this study mostly came from the analysis of T2DM patients. Therefore, the results need to be cautiously interpreted.

In conclusion, this study suggests that serum cortisol secretion is directly associated with the presence of microalbuminuria in patients with T2DM and patients with prediabetes. Higher levels of cortisol, even in the normal range, may be related with the development of microalbuminuria. Further researches are needed to clarify whether there is a cause-and-effect relationship between cortisol and the development of microalbuminuria.

## Supplementary Material

Supplementary tables.

## Figures and Tables

**Table 1:** Clinical characteristics of subjects

Characteristics	Normoalbuminuria	Microalbuminuria	All	*P* value
	N = 120	N = 91	N = 211	
**Basic characteristics**				
Age (years)	56.0 (16.0)	55.0 (25.0)	56.0 (21)	0.564
Female/male (%)	69/51 (57.5/42.5)	43/48 (47.3/52.7)	112/99 (53.1/46.9)	0.140
Diabetes/Prediabetes (%)	110/10 (91.7/8.3)	88/3 (96.7/3.3)	198/13 (93.8/6.2)	0.132
Duration of diabetes (years)	3.0 (8.0)	3.0 (7.0)	3.0 (10)	0.923
Previous diabetes treatment (%)				0.422
None	46 (41.8)	35 (39.8)	81 (40.9)	
OHA	50 (45.5)	39 (44.3)	89 (44.9)	
One type	9 (8.2)	12 (13.6)	21 (10.6)	
Two types	22 (20.0)	16 (14.5)	38 (19.2)	
Three or over three types	19 (17.3)	11 (12.5)	30 (15.2)	
Insulin alone	8 (7.3)	4 (4.5)	12 (6.1)	
Both OHA and insulin	6 (5.5)	10 (11.4)	16 (8.1)	
Past history of hypertension	73 (60.8)	74 (81.3)	147 (69.7)	0.001^*^
Previous hypertension treatment (%)	73	74	147	0.927
None	28 (38.4)	29 (39.2)	57 (38.8)	
One type	14 (19.2)	13 (17.6)	27 (18.4)	
Two types	17 (23.3)	15 (20.3)	32 (21.8)	
Three or over three types	14 (19.2)	17 (23.0)	31 (21.1)	
BMI (kg/m^2^)	26.6 (7.1)	27.1 (6.4)	27.0 (6.9)	0.471
Smoking	21 (17.8)	26 (28.6)	47 (22.5)	0.064
**Cortisol and ACTH**				
Cortisol (nmol/L)				
8:00 h	367.0 (173.8)	397.0 (193.0)	364.0 (143.0)	0.012^*^
16:00 h	205.0 (159.2)	268.0 (168.5)	206.5 (186.0)	0.001^*^
0:00 h	83.9 (95.7)	92.4 (128.7)	83.3 (105.6)	0.655
ACTH (pmol/L)				
8:00 h	4.77 (3.90)	5.19 (3.62)	5.04 (4.26)	0.075
16:00 h	3.26 (2.04)	3.52 (2.19)	3.46 (1.90)	0.437
0:00 h	1.88 (1.72)	1.99 (1.48)	1.97 (1.53)	0.798
Lack of circadian rhythm	20 (20.2)	22 (30.6)	42 (24.6)	0.120
**Urinary albumin excretion**				
Albumin excretion rate (mg/24h)	12.18 (13.22)	71.19 (78.34)	20.68 (47.83)	< 0.001^*^
Urinary total protein (mg/24h)	96.22 (63.04)	308.0 (1260.6)	121.00 (153.65)	< 0.001^*^
Urinary albumin (mg/L)	5.99 (7.20)	39.22 (36.04)	10.16 (20.84)	< 0.001^*^
**Complications**				
Retinopathy	2 (1.9)	8 (9.2)	10 (5.1)	0.045^*^
Peripheral neuropathy	20 (18.3)	11 (12.5)	31 (15.7)	0.262
Foot ulcer	1 (0.9)	1 (1.1)	2 (1.0)	0.879
**Metabolic and laboratory measurements**				
HbA1c (%)	7.8 (2.1)	8.4 (2.5)	8.1 (2.3)	0.053
FPG (mmol/L)	7.64 (3.48)	8.03 (3.69)	7.81 (3.57)	0.431
2h PG (mmol/L)	12.81 (5.48)	13.39 (4.95)	13.03 (5.27)	0.583
Fasting C-Peptide (μg/L)	2.98 (1.36)	3.53 (2.34)	3.23 (1.88)	0.121
2h C-Peptide (μg/L)	6.45 (3.17)	7.51 (8.47)	6.93 (6.16)	0.387
Systolic BP (mm Hg)	138.9 (24.3)	155.8 (28.5)	146.2 (27.4)	< 0.001^*^
Diastolic BP (mm Hg)	83.9 (13.9)	90.8 (18.3)	86.8 (16.2)	0.003^*^
HDL-C (mmol/L)	1.05 (0.27)	1.03 (0.58)	1.04 (0.43)	0.764
LDL-C (mmol/L)	3.01 (0.93)	3.12 (1.13)	3.05 (1.02)	0.449
Triglycerides (mmol/L)	1.68 (1.30)	1.84 (1.29)	1.88 (1.16)	0.179
Total cholesterol (mmol/L)	4.70 (1.12)	4.94 (1.36)	4.80 (1.23)	0.164
ApoA1 (g/L)	1.16 (0.32)	1.18 (0.34)	1.11 (0.25)	0.769
ApoB (g/L)	0.96 (0.27)	1.01 (0.32)	0.99 (0.29)	0.426
Uric acid (μmol/L)	396.1 (120.5)	407.5 (114.0)	401.1 (122.8)	0.513
hs-CRP (mg/L)	1.4 (2.2)	2.6 (4.5)	3.8 (29.4)	0.034^*^
Albumin (g/L)	41.0 (5.9)	39.8 (6.7)	39.6 (7.5)	0.108
eGFR (mL/min/1.73 m^2^)	88.60 (24.75)	74.45 (35.26)	82.58 (30.42)	0.002^*^

Continuous data are presented as mean (standard deviation) or median (interquartile range), categorical data as number (percentage); N, number of patients; OHA, oral hypoglycemic agent; BMI, body mass index; ACTH, adrenocorticotropic hormone; FPG, free plasma glucose; PG, plasma glucose; HbA1c, glycated hemoglobin; BP, blood pressure; HDL-C, high-density lipoprotein cholesterol; LDL-C, low-density lipoprotein cholesterol; Apo, apolipoprotein; hs-CRP, high sensitivity C-reactive protein; eGFR, estimated glomerular filtration rate; ^*^P-value < 0.05.

**Table 2:** Factors correlated with microalbuminuria

Characteristics	Univariate		Multivariate		Multivariate	
	β	*P*	β	*P*	β	*P*
Past history of hypertension: n (%)	1.031	0.002^*^	-	-	-	-
Smoking: n (%)	0.614	0.066	0.682	0.088	0.453	0.324
Diabetic retinopathy: n (%)	1.690	0.036^*^	1.595	0.083	0.049	0.965
HbA1c (%)	0.119	0.055	0.076	0.160	0.099	0.238
Systolic BP (mm Hg)	0.025	< 0.001^*^	0.022	0.015^*^	0.036	0.001^*^
Diastolic BP (mm Hg)	0.027	0.003^*^	0.014	0.798	-0.003	0.841
Cortisol at 8:00 h (nmol/L)	0.003	0.006^*^	0.004	0.005^*^	-	-
Cortisol at 16:00 h (nmol/L)	0.004	0.002^*^	-	-	0.005	0.001^*^
eGFR (mL/min/1.73 m^2^)	-0.016	0.001^*^	-0.017	0.005^*^	-0.008	0.210

Cortisol level at 8:00 h and at 16:00 h were put in multivariate analysis separately to decrease the possible bias caused by combination enhanced effects. HbA1c, glycated hemoglobin; BP, blood pressure; eGFR, estimated glomerular filtration rate; ^*^P-value < 0.05.

**Table 3:** Correlation between urinary albumin excretion rate (mg/24h) and demographic and metabolic parameters

Variable	Correlation coefficient	*P*
Past history of hypertension: n (%)	0.313	0.001^*^
Smoking: n (%)	0.261	0.008^*^
Diabetic retinopathy: n (%)	0.246	0.013^*^
HbA1c (%)	0.231	0.022^*^
Systolic BP (mm Hg)	0.387	< 0.001^*^
Diastolic BP (mm Hg)	0.199	0.042^*^
Cortisol at 8:00 h (nmol/L)	0.183	0.061
Cortisol at 16:00 h (nmol/L)	0.224	0.046^*^
Cortisol at 0:00 h (nmol/L)	0.149	0.189
eGFR (mL/min/1.73 m^2^)	-0.299	0.002^*^

HbA1c, glycated hemoglobin; BP, blood pressure; eGFR, estimated glomerular filtration rate; ^*^P-value < 0.05.
