# Supplementary material for: The Association Of Serum Cortisol Level With Microalbuminuria In Patients With Type 2 Diabetes And Prediabetes
Source: Int J Med Sci. 2020 Oct 18;17(18):2998–3004. doi: 10.7150/ijms.48742 (PMC7646104; doi:10.7150/ijms.48742)
Supplement: Supplementary file 1 — Supplementary tables. [file ijmsv17p2998s1.pdf]

Supplementary Table 1 Correlation between urinary protein levels (mg/24h) and demographic and metabolic parameters

Variable	Correlation coefficient	*P*
Past history of hypertension: n (%)	0.198	0.006*
Smoking: n (%)	0.097	0.185
Diabetic retinopathy: n (%)	0.191	0.011*
HbA1c (%)	0.049	0.510
Systolic BP (mm Hg)	0.255	< 0.001*
Diastolic BP (mm Hg)	0.082	0.261
Cortisol at 8:00 h (nmol/L)	0.112	0.126
Cortisol at 16:00 h (nmol/L)	0.090	0.274
Cortisol at 0:00 h (nmol/L)	0.043	0.607
eGFR (mL/min/1.73 m^2)	-0.324	< 0.001*

HbA1c, glycated hemoglobin; BP, blood pressure; eGFR, estimated glomerular filtration rate; *P-value < 0.05.

Supplementary Table 2 Correlation between cortisol level at 8:00 h (nmol/L) and demographic and metabolic parameters

Variable	Correlation coefficient	*P*
Age	0.034	0.620
Gender	-0.031	0.659
Past history of hypertension: n (%)	0.014	0.840
BMI (kg/m^2)	0.098	0.181

Smoking: n (%)	0.035	0.613
HbA1c (%)	0.143	0.042*
FPG (mmol/L)	0.129	0.065
Systolic BP (mm Hg)	-0.008	0.910
Diastolic BP (mm Hg)	-0.045	0.520
eGFR (mL/min/1.73 m^2)	0.011	0.870
Urinary total protein (mg/24h)	0.112	0.126
Albumin excretion rate (mg/24h)	0.183	0.061
Urinary albumin (mg/L)	0.190	0.028*

BMI, body mass index; HbA1c, glycated hemoglobin; FPG, free plasma glucose; BP, blood pressure; eGFR, estimated glomerular filtration rate; *P-value < 0.05.

Supplementary Table 3 Correlation between cortisol level at 16:00 h (nmol/L) and demographic and metabolic parameters

Variable	Correlation coefficient	*P*
Age	-0.093	0.226
Gender	-0.140	0.069
Past history of hypertension: n (%)	0.018	0.821
BMI (kg/m^2)	0.088	0.151
Smoking: n (%)	0.069	0.372
HbA1c (%)	0.122	0.126
FPG (mmol/L)	0.186	0.017*
Systolic BP (mm Hg)	-0.003	0.966
Diastolic BP (mm Hg)	0.018	0.819

eGFR (mL/min/1.73 m^2)	0.045	0.564
Urinary total protein (mg/24h)	0.090	0.150
Albumin excretion rate (mg/24h)	0.224	0.046*
Urinary albumin (mg/L)	0.228	0.020*

BMI, body mass index; HbA1c, glycated hemoglobin; FPG, free plasma glucose; BP, blood pressure; eGFR, estimated glomerular filtration rate; *P-value < 0.05.
